# *In vitro* and *in vivo* validation studies of optimized iron oxide nanoparticles carrying targeting ligands for a new therapeutic strategy in head and neck cancers

**DOI:** 10.1039/d5na00361j

**Published:** 2025-09-03

**Authors:** Sonia Furgiuele, Thomas Gevart, Barbara Freis, Maria Los Angeles Ramirez, Géraldine Descamps, Sébastien Boutry, Lionel Larbanoix, Dorianne Sant'Angelo, Anne Trelcat, Sven Saussez, Sylvie Bégin-Colin, Fabrice Journe, Sophie Laurent

**Affiliations:** a Department of Human Anatomy and Experimental Oncology, Faculty of Medicine, Research Institute for Health Sciences and Technology, University of Mons (UMONS) Avenue du Champ de Mars, 8 B7000 Mons Belgium sonia.furgiuele@umons.ac.be geraldine.descamps@umons.ac.be anne.trelcat@umons.ac.be sven.saussez@umons.ac.be fabrice.journe@umons.ac.be; b General, Organic and Biomedical Chemistry Unit, NMR and Molecular Imaging Laboratory, University of Mons B-7000 Mons Belgium barbara.freis@ipcms.unistra.fr thomas.gevart@umons.ac.be sophie.laurent@umons.ac.be; c University of Strasbourg, CNRS, Institute of Physics and Chemistry of Materials of Strasbourg, UMR7504 67034 Strasbourg Cedex France sylvie.begin@unistra.fr; d Center for Microscopy and Molecular Imaging B-6041 Gosselies Belgium sebastien.boutry@umons.ac.be lionel.larbanoix@umons.ac.be; e Department of Otolaryngology and Head and Neck Surgery, CHU Saint-Pierre 1000 Brussels Belgium; f Laboratory of Clinical and Experimental Oncology, Institute Jules Bordet, Université Libre de Bruxelles (ULB) 1000 Brussels Belgium

## Abstract

Iron oxide nanoparticles (IONPs) are increasingly used in the biomedical field. Indeed, they can improve patient diagnosis, as they are excellent *T*_2_ contrast agents for magnetic resonance imaging (MRI), and they can be considered a therapeutic and radiosensitizing agent for cancer by influencing the redox balance. However, to achieve specific accumulation of nanoparticles in the tumor, active targeting with specific ligands is required. In this context, we have developed IONPs that would specifically target head and neck cancer (HNC) cells. First, we optimized IONP synthesis and produced dendronized IONPs that were coupled with the targeting ligand cRGD (@D + cRGD IONPs) or peptide 22 (@D + P22 IONPs). The former is a tripeptide with affinity for integrins while the latter is a dodecapeptide analog of GE11, an EGF (epidermal growth factor) derived polypeptide with affinity for EGFR. EGFR is overexpressed in these HNC cells. Next, we highlighted the interest of using @D + P22 IONPs in order to enhance internalization of IONPs *in vitro*. Furthermore, we evaluated the biodistribution of IONPs *in vivo* and showed by MRI an immediate *T*_2_ contrast in the liver and kidney, whatever the type of IONP. Finally, we developed an *in vivo* model of mice with FaDu xenografts and showed by 
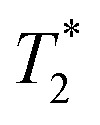
 MRI a tendency for higher accumulation of @D + P22 IONPs (10.1% decrease of *T*_2_ calculated in the tumoral region by 
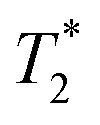
 measurement) compared to @D IONPs (0.15%) within the tumors. These preliminary results are encouraging and require further investigations, but they suggest the potential interest of using this IONP model for targeting EGFR-positive tumors.

## Abstract 2

Dendronized iron oxide nanoparticles vectorized with peptide P22 show enhanced uptake in head and neck cancer cells and promising accumulation in tumors for targeted MRI theranostics.

## Graphical abstract

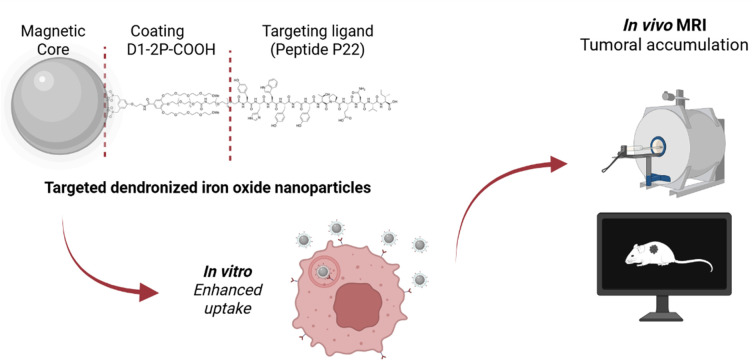

## 1. Introduction

Over the past decade, nanomedicine has developed significantly in the field of oncology. This medicine uses nanometric materials, like nanoparticles (NPs), for both diagnostic and therapeutic purposes, and is therefore known as a theranostic agent. In head and neck cancers (HNCs), research is aimed at improving diagnostic and prognostic tools and increasing the efficacy of therapies.^1^ Nanomedicine could be a game-changer for these cancers. HNC remains one of the most widespread cancers worldwide, ranking in 6th place.^2^ Despite advances in therapeutic strategies, including surgery and concomitant radio-chemotherapy, HNCs are still associated with an unfavorable prognosis with a 5-year survival rate of around 50% and recurrences occurring in 40–60% of treated patients.^3^ Therefore, it is essential to find new treatment approaches, and the use of NPs should be effective in this goal.

There are different classes of NPs including inorganic NPs such as gold (GNPs), iron oxide (IONPs), and silica NPs, which are always under evaluation in clinical and translational cancer studies. These NPs are characterized by different sizes, shapes and structures giving them specificity for targeting certain cells. GNPs and IONPs are among the most studied nanoparticles, and IONPs have already been accepted by the Food and Drug Administration (FDA) for use in nanomedicine applications as magnetic resonance imaging (MRI) contrast agents or as nano-heaters in magnetic hyperthermia treatment.^4,5^ Indeed, they present superparamagnetic properties allowing their utilization as effective and excellent contrast agents for MRI, which is a non-invasive imaging technique.^6–9^ Therefore, these IONPs are of particular interest for tumor detection and diagnosis. IONPs also have therapeutic prospects in cancers thanks to their involvement in magnetic hyperthermia and in photothermal and photodynamic therapy, leading to improved therapeutic settings.^5,10–12^ They are also being examined as radio-sensitizing factors due to iron ion release and ROS production after internalization of these NPs into cancer cells.^13^

To refine the targeting specificity of NPs, many studies suggest grafting vectors such as peptides or antibodies. The choice of the ligand is an important step in the synthesis of vectorized NPs. Indeed, the size of the targeting molecule must not interfere with the functionality of the nanomaterial. For example, the use of peptides has less impact on the size modifications of NPs than the use of antibodies. A very relevant peptide to increase internalization of NPs into cancer cells is RGD. This peptide is composed of 3 amino acids with an Arg-Gly-Asp motif and has specificity for the integrins αvβ3 and αvβ5 overexpressed by cancer cells and in particular in HNCs.^14,15^ Otherwise, the overexpression of epidermal growth factor receptor (EGFR) is probably the most relevant target for these cancers. Indeed, this receptor is upregulated in 80% of HNCs^16^ and is strongly involved in tumorigenesis and in mechanisms of resistance to treatment. In this context, the low molecular weight dodecapeptide GE11 (YHWYGYTPQNVI) has been demonstrated to have a high affinity for EGFR.^17^ Then, many analogs of the GE11 peptide were screened to search for a peptide with the highest affinity for EGFR using triple negative breast cancer cells (MDA-MB-231 line) overexpressing this receptor as a model. Hossein-Nejad-Ariani *et al.* demonstrated that the peptide analog 22 (YHWYGYTPENVI) (P22) exhibits higher uptake in cancer cells than the initial peptide GE11 (123-fold and 47-fold in MDA-MB-468 and MDA-MB-231 cell lines, respectively).^18^ P22 would therefore be the best candidate for targeting all types of cancers overexpressing EGFR, like HNCs. Furthermore, we previously optimized the coupling reaction of P22 at the surface of dendronized IONPs^19^ (@D IONPs).

In this work, we first validated the overexpression of EGFR in a panel of 6 HNC cell lines and highlighted the advantage of vectorization of @D IONPs to EGFR for internalization into HNC cells (FaDu and 93VU-147T lines). Then, we evaluated the biodistribution of IONPs in a mouse model with and without tumor xenografts by MRI tracking, ICP-AES analysis, and immunofluorescence.

## 2. Materials and methods

### 2.1. NP synthesis

IONPs were synthesized by thermal decomposition of an iron stearate precursor in an organic solvent with a high boiling point and a surfactant according to a reported procedure.^20,21^ In a two-neck round-bottom-flask of 100 mL, 2.2 mmol of FeSt_3_ (1.99 g) (purity min 60% of stearic acid, 5.8% to 7% of iron and maximum 10% of free acid, TCI, Tokyo, Japan) and 4.4 mmol of oleic acid (OA) (1.24 g) (purity 99%, Alfa Aesar, Heysham, United Kingdom) were mixed with dioctyl ether (16.2 g, 20 mL) (purity 99%, Sigma Aldrich, St. Louis, MI, USA). The mixture was first heated to 120 °C (heating device temperature: 130 °C) for 60 min to dissolve the reagents in dioctyl ether. After this step, the condenser was connected to the flask and the mixture was heated up to 290 °C (heating device temperature: 320 °C) with a 5 °C min^−1^ ramp. The mixture was refluxed at 290 °C for 120 min. The obtained black NP suspension was cooled down to 100 °C to proceed to the washing steps. 10 mL of chloroform (purity 99%, Carlo Erba, France) were added to the IONP suspension at a temperature of around 100 °C and then this suspension was introduced in a flask containing 400 mL of acetone (purity 99.8%, Carlo Erba, France). For the washing of the 12 nm suspension, the mixture was heated at 60 °C for 1 h under mechanical stirring using a thermal bath. The IONPs were collected with a magnet and the supernatant was discarded. The collected IONPs were redispersed in 50 mL of chloroform. Then, 400 mL of acetone were added again to proceed to a second washing step and the mixture was once more heated at 60 °C for 45 min under mechanical stirring. At the end, the IONPs were collected with a magnet and the supernatant was discarded. Finally, the IONPs were resuspended in 40 mL of tetrahydrofuran (THF) (purity 99.5%, Carlo Erba, France) for their storage or further utilization. Transmission electron microscopy analysis revealed a spherical core size of the IONPs of 12.8 nm. The physicochemical properties of these nanoparticles were described previously (Freis *et al.*^19^).

### 2.2. NP dendronization

**Fig. 1:**
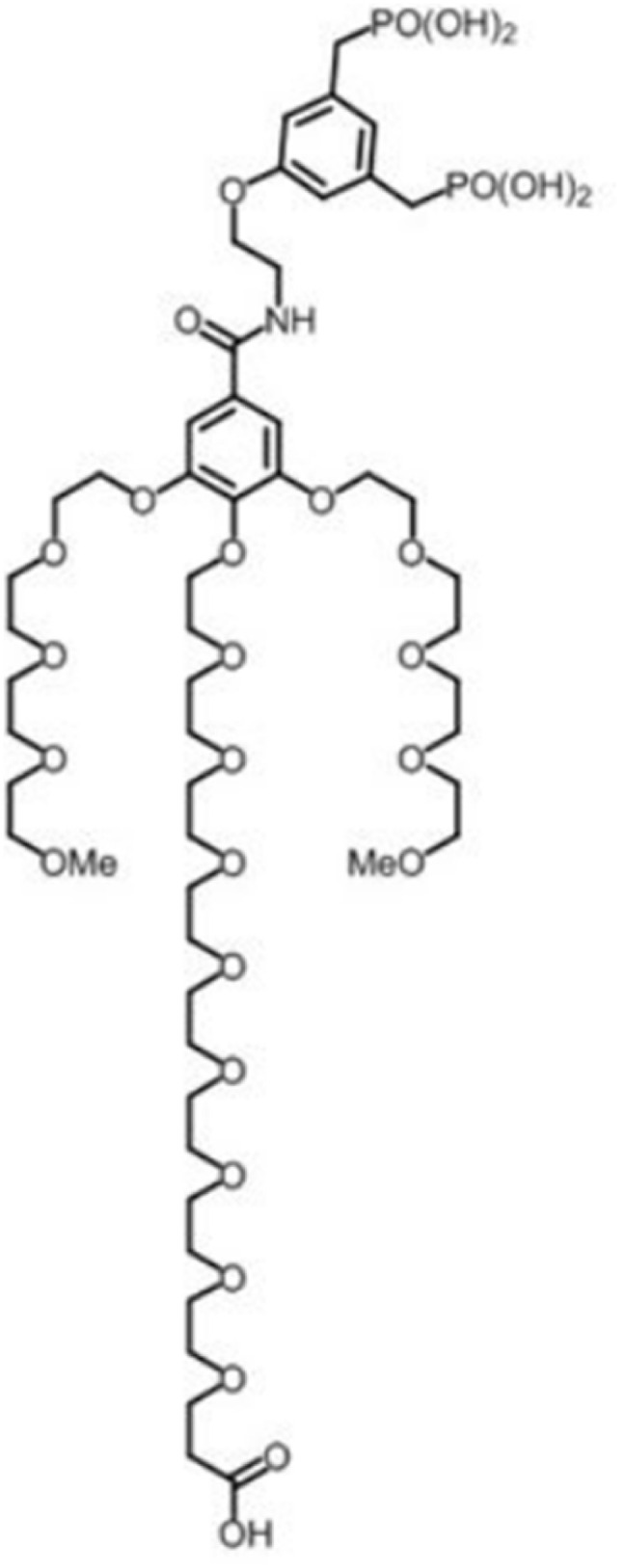
Molecular structure of dendron D1-2P-CO2H from SuperBranche® (Strasbourg, France).

For the dendronization of IONPs, ligand exchange between OA and the dendron (Fig. 1, D1-2P, Superbranche, Strasbourg, France^22^) was performed in THF as in previously reported publications.^19,23^ Briefly, 5 mL of IONPs@OA at 1 mg Fe per mL were stirred for 24 h with 7 mg of dendron. To maximize the ligand exchange and remove most of the OA molecules, the suspension was then purified by ultrafiltration. After that, 5 mg of dendron were added to the mixture for another 24 h of stirring. The suspension was mixed with hexane (volume ratio 1/3 : 2/3) (Carlo Erba, France) to precipitate the @D IONPs. The suspension was centrifuged at 8000 rpm for 5 min and the supernatant was discarded. Finally, the DNPs were collected and dispersed in deionized water. The water suspension was purified 3 times with a 50 mL Amicon stirred cell ultrafiltering unit. Dynamic light scattering (DLS) analysis indicated a mean hydrodynamic diameter of 18.7 nm for the dendronized IONPs.

### 2.3. NP vectorization to EGFR

Then, two protocols were used for the coupling of the targeting ligand peptide 22 (P22) (purity 98.7%, polypeptide, Strasbourg, France) and cRGD (Eurogentec, Angers, France). These two protocols are from ref. 19.

#### 2.3.1. Protocol 1

The DNP suspension at a concentration of 0.5 mg Fe per mL is mixed with 1-ethyl-3-(3′-dimethylaminopropyl)carbodiimide hydrochloride (EDC-HCl, purity 99%, Carl Roth, Karlsruhe, Germany) (20 molar excess compared to carboxylate groups present on dendron molecules) for 1 to 2 min and 0.6 P22 molar equivalent (compared to dendron molecules) is directly added to the suspension and stirred overnight. At the end, the supernatant was recovered by centrifugation (10 min at 8000 rpm) in centrifugal filter units (Amicon®, MW cut off: 30 kDa, Merck Millipore, Germany). Then, three washes with MilliQ water were done and the washes were recovered for unbound P22 quantification.

#### 2.3.2. Protocol 2

The DNP suspension was initially buffer-exchanged with HEPES buffer 0.1 M, pH 6.5 (purity 99.5%, Sigma Aldrich, St. Louis, MI, USA) in a centrifugal filter unit (Amicon®, MW cut off: 30 kDa; 10 min at 8000 rpm); the filtrate was resuspended in an appropriate volume to reach a final concentration of 5 mg Fe per mL in HEPES (99.5%, Sigma-Aldrich, France). Then, the suspension was mixed with EDC (20 molar excess compared to carboxylate groups present on dendron molecules) for 10 min and then *N*-hydroxy sulfosuccinimide (sulfo-NHS, purity 98%, Sigma Aldrich, St. Louis, MI, USA) (20 molar excess compared to carboxylate groups present on the dendron molecule) was added and mixed for an additional 20 min. Upon the sulfo-NHS addition, the volume was increased to reach a final iron concentration of 0.5 mg mL^−1^ in HEPES buffer. The activated DNPs were rinsed with 0.1 M carbonate buffer, pH 9.2, (prepared from sodium carbonate and sodium hydrogenocarbonate from Sigma Aldrich, France) in a centrifugal filter unit (10 min at 8000 rpm) to eliminate excess EDC, sulfo-NHS and other possible reaction intermediates. The filtrate was rapidly recovered and mixed with 1.2 P22 molar equivalents (compared to dendron molecules) in carbonate buffer. The volume of the reaction was adjusted with carbonate buffer to reach a final iron concentration of 0.5 mg mL^−1^. The reaction was left overnight at room temperature under magnetic stirring. At the end, the supernatant was recovered by centrifugation (10 min at 8000 rpm) in centrifugal filter units (Amicon®, MW cut off: 30 kDa). Then, three washes with MilliQ water were done and the washes were recovered for unbound P22 quantification.

DLS measurements showed that the mean hydrodynamic diameter increased to 20.6 nm for nanoparticles prepared with Protocol 1 and to 21.7 nm for Protocol 2, while the zeta potential shifted from −22 mV for dendronized IONPs to −29 (Protocol 1) and −42 mV (Protocol 2), confirming surface modification upon ligand conjugation.^19^

### 2.4. Cell cultures

Six HNC cell lines, 3 HPV-negative (FaDu, Detroit 562 and UPCI-SCC-131) and 3 HPV-positive (93-VU-147T, UM-SCC-47 and UPCI-SCC-154), were used to evaluate EGFR expression. FaDu (ATCC HTB-43) was derived from the hypopharyngeal carcinoma, Detroit 562 (ATCC CCL-138) was derived from a metastatic site in the pleural fluid of a patient with a pharyngeal carcinoma, UPCI-SCC-131 (DMCZ ACC 668) was established from a squamous cell carcinoma of the oral cavity, 93-VU-147T (gifted from Dr De Winter, University Medical Center of Amsterdam) was derived from oral cavity carcinoma, UM-SCC-47 (CVCL_7759) was established from a tongue squamous cell carcinoma, and UPCI-SCC-154 (DSMZ ACC 669) was derived from the primary tumor of the tongue. All cells were grown in DMEM 4.5 g per L glucose without l-glutamine (Westburg BE12-614F, Leusden, The Netherlands), supplemented with 10% heat-inactivated fetal bovine serum (FBS Premium South America, PAN BIO-TECH, Aidenbach, Germany), 2% l-glutamine (200 mM, Gibco, Thermo Fisher Scientific, Waltham, MA, USA), and 1% penicillin/streptomycin (10 000 U mL^−1^/10 000 μg mL^−1^, Gibco, Thermo Fisher Scientific, Waltham, MA, USA). Cultures were maintained by the replacement of the medium and kept at 37 °C in a humidified 95% air and 5% CO_2_ atmosphere. Subcultures were obtained using trypsin–EDTA when cells reached 80% of confluence. The cultures were confirmed to be free of mycoplasma contamination using PCR-based detection.

### 2.5. Colorimetric iron dosing method by Perls' Prussian blue reaction

FaDu and 93-VU cell lines were seeded in 6-well plates in order to be at 80% of confluence after 48 h. After 24 h, cells were exposed for 24 h to 50 μg per mL NPs diluted in complete DMEM. Then, evaluation of NP internalization was performed using the Prussian blue reaction.^24^ First, cells were counted and washed 3 times with DPBS without calcium and magnesium (Thermo Fisher Scientific, Waltham, MA, USA) and centrifuged into pellets. Then, 200 μL of 5 M HCl (37%, 5 mol L^−1^, ChemLab, Zedelgem, Belgium) were added to the pellets in order to lyse the cell membrane and they were placed for 24 h at 37 °C on a heating block. Then, the samples were briefly centrifuged and 100 μL of 5% potassium ferrocyanide trihydrate (Acros Organics, Thermo Fisher Scientific, Geel, Belgium) were added to the supernatant. Samples were mixed for 15 min. At the same time, a calibration curve was obtained. A range of iron concentrations (0 to 0.3 μmol mL^−1^) was prepared and 100 μL of 5 M HCl and 100 μL of potassium ferrocyanide trihydrate were added. Finally, colorimetric absorbance (optical density) was measured at 650 nm with a microplate reader (VERSA max-SoftMax Pro, VWR, Leuven, Belgium), and referring to the calibration curve, the amount of iron internalized per cell (pg iron per cell) was calculated.

### 2.6. Western blotting of EGFR

Proteins were extracted by cell lysis using Mammalian Protein Extraction Reagent and Protease & Phosphatase Inhibitor Cocktail 100× (both from Pierce, Rockford, IL, USA) and dosed using a BCA Pierce Protein Assay Kit (ThermoFisher Scientific, Rockford, IL, USA). Then, 20 μg of proteins were charged on a 4–20% polyacrylamide gel (Bio-Rad Laboratories, Hercules, CA, USA) and migrated at 120 volt for 1 h. The transfer on the nitrocellulose membrane was performed using an iBlot 2 Dry Blotting system (ThermoFisher Scientific). The primary anti-EGF receptor D38B1 (rabbit, dilution 1 : 1000) (Cell Signaling Technology #4267) was diluted in TBS-Tween 0.1% BSA 5%. Detection was performed using a Novex™ ECL Chemiluminescent Substrate Reagent Kit (Invitrogen, Carlsbad, CA, USA). The revelation was performed with the chemiluminescence imaging system Fusion FX (Vilber) and quantified using Image J.

### 2.7. *In vitro* immunofluorescence staining

HNC cell lines were seeded on coverslips in 12- or 24-well plates in order to reach 80% confluence after 48 h. After 24 h, cells were exposed for 24 h to 50 μg per mL NPs diluted in complete DMEM. Then, cells were fixed for 15 min (10 min at 4 °C followed by 5 min at room temperature) with 4% paraformaldehyde (PAF) (Sigma-Aldrich, St. Louis, MI, USA) in PBS, then rinsed with PBS 1× and washed 3 times for 5 min with 0.1% Triton™ X-100 (Sigma-Aldrich, Merck, Overijse, Belgium) in PBS 1×. Next, cells were incubated with 0.05% casein, a blocking solution, for 30 min at room temperature. Then, the cells were incubated overnight at 4 °C with primary antibodies, an anti-polyethylene glycol antibody [PEG-B-47] (Abcam, Cambridge, UK) or an anti-EGFR [MA5-13269] or [MA5-13070] (Thermo Fisher Scientific, Waltham, MA, USA) both at 1/100 dilution. After 3 washes with 0.1% Triton™ X-100 in PBS 1×, cells were incubated with secondary antibodies, a goat anti-rabbit IgG (H + L) (Highly Cross-Absorbed Secondary Antibody, Alexa Fluor Plus 488, A31627, Thermo Fisher Scientific, Waltham, MA, USA) or a goat anti-mouse IgG (H + L) (Highly Cross-Absorbed Secondary Antibody, Alexa Fluor Plus 555, A32727, Thermo Fisher Scientific, Waltham, MA, USA) at 1/500 dilution for 1 h at room temperature, depending on the primary antibody used. The coverslips were then rinsed with 0.1% Triton™ X-100 in PBS 1× followed by distilled water before being mounted on slides with VectaShield-DAPI (VectaShield, Vector Laboratories, Newark, CA, USA). Once dried, the slides could be observed under a confocal microscope (Nikon Ti2 A1RHD25, Tokyo, Japan).

### 2.8. Apoptosis evaluation

FaDu and 93-VU cell lines were seeded in 6-well plates in order to be at 80% confluence after 48 h. After 24 h, the cells were exposed for 24 h to 50 μg per mL NPs diluted in complete DMEM. Then, evaluation of NP-induced apoptosis was performed using an Annexin V & Dead Cell Kit (Muse® Annexin V & Dead Cell Kit, Luminex, USA). First, cells are removed by trypsinization, and the cell suspension is added to the culture medium that was previously collected. Then, this mixture consisting of dead and living cells is counted in order to produce a cell suspension at 5 × 10^5^ cells per mL. 100 μL of this cell suspension are taken and mixed with 100 μL of reagent from the kit. The solution is vortexed and incubated for 20 min away from light and at room temperature before being analyzed using a Muse flow cytometer (Guava® Muse® Cell Analyzer, Luminex, The Netherlands).

### 2.9. Intracellular ROS evaluation

Detection of ROS in FaDu and 93-VU cell lines after NP exposition was performed using a Muse® Oxidative Stress Kit (Luminex, MCH100111, Austin, TX, USA) based on the use of the permeable reagent dihydroethidium (DHE). Cells were prepared in 1× assay buffer (Luminex, 4700-1330) at 1.5 × 10^6^ cells per mL. Then, 10 μL of cell suspension were incubated for 30 min at 37 °C with 190 μL of the Muse® Oxidative Stress Reagent (Luminex, 4700-1665, Austin, TX, USA) prediluted 800× with 1× assay buffer. Finally, the cell suspension was mixed thoroughly and run on a Guava® Muse Cell Analyzer according to the manufacturer's recommendations to detect ROS (−) and ROS (+) cells.

### 2.10. NP biodistribution *in vivo*

*In vivo* experiments were conducted at the Center for Microscopy and Molecular Imaging (CMMI, Gosselies, Belgium). All animal experiments were approved by and performed in accordance with the guidelines of the ethics committee of the CMMI (number LA1500589) under the specific agreement number 2021-02. Sixteen nude mice and 23 BalbC mice (all 6 weeks-old females) were purchased from Charles River (Saint-Germain-Nuelles, FR). First, 8 BalbC mice were used to evaluate the IONP biodistribution after intravenous (IV) injection in the tail vein at 45 μmol Fe per kg (@D IONPs *n* = 5 and @D + P22 IONPs *n* = 3), up to 1 month post-injection (including an MRI follow-up and a subsequent *ex vivo* ICP-AES iron dosage in selected organs; see 2.12). Fifteen other BalbC mice were used as the control (*n* = 3) and for biodistribution studies at shorter time points (24 h: @D IONPs *n* = 3 and @D + P22 IONPs *n* = 3 and 4 h: @D IONPs *n* = 3 and @D + P22 IONPs *n* = 3) with ICP-AES only (see 2.12). At each time point (4 h, 24 h, 1 week and 1 month), mice were sacrificed and their kidneys, liver, spleen and femoral bone were harvested and frozen.

Next, tumors were established in 16 nude mice by subcutaneously injecting 2.5 or 5 × 10^6^ FaDu cells with or without Matrigel (R&D Systems, Minneapolis, MN, USA) into the left lower flank. When the tumor reached a volume of ∼400 mm^3^, the mice were examined for biodistribution of NPs in the tumor context post-IV injection (60 μmol Fe per kg @D IONPs (*n* = 7) or @D + P22 IONPs (*n* = 6) + 3 non-injected controls). In preliminary MRI experiments, one mouse of each injected group was followed up to one day post-injection, with MRI visualization at 2 h 30 min and at 24 h. For subsequent *ex vivo* ICP-AES measurements in tumors and selected organs (see 2.12), all injected mice were sacrificed 24 h post-injection, except one mouse in each group, which was sacrificed one-week post-injection.

### 2.11. MRI

Mice were anesthetized with isoflurane vaporized in oxygen (Zoetis from Alcyon SA) and placed in a cradle adapted for mouse body imaging. Animal temperature was maintained by warm water circulating in a blanket during the whole anesthesia period, and the respiratory rate was monitored (30 breaths per min). Mice were intravenously injected with @D IONPs or @D + P22 IONPS (45 μmol Fe per kg for main biodistribution/imaging experiments in BalbC mice and 60 μmol Fe per kg for molecular biodistribution/imaging experiments in tumor-bearing nude mice (to maximize the dose for molecular MRI application, in the ethical limits of IV injectable volume in mice) through a catheter (30 G needle) placed in the tail vein. MRI was performed with a Bruker Biospec 9.4T scanner, using a 40 mm volume coil dedicated to mouse body imaging. Main MRI sequences for biodistribution/MRI experiments were fat-suppressed coronal spin-echo RARE covering the whole body with 1 mm slices; slightly *T*_2_-weighted (TR/TEeff: 2000/17 ms, 129 × 121 microns resolution, RARE factor 4, NEX = 5, acquisition time: 15 min 30 s) or strongly *T*_2_-weighted (TR/TEeff: 4270/75 ms, 206 × 208 microns resolution, RARE factor 16, NEX = 4, acquisition time: 3 min 42 s). A *T*_2_-weighted axial fat-suppressed spin-echo RARE sequence was also used for attempting to get indicative images of the bladder (TR/TEeff: 7730/52 ms, 125 × 135 microns resolution, RARE factor 8, NEX = 4, 1 mm slices, acquisition time 9 min 16 s). Main MRI sequences for molecular biodistribution/imaging experiments in tumor-bearing nude mice were *T*_2_-weighted anatomical fat-suppressed spin-echo RARE with 1 mm slices in axial or sagittal orientation, aiming at tumor scouting (TR/TEeff: 2500/40.2 ms, NEX = 4, 167 × 156 microns in sagittal orientation (2 min 40 s acquisition time) and 125 × 125 microns in axial orientation (3 min 20 s acquisition time)), and 
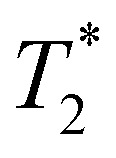
-weighted multi-echo gradient echo (MGE) for more sensitive tumor-focused imaging of IONP distribution; briefly, magnetic field homogeneity (shim) was optimized on a volume covering the tumor with a standard process: after fieldmap acquisition using the MAPSHIM tool, a water peak was acquired with this volume using a PRESS-waterline spectroscopy sequence, and its width (measured with the CalcLineWidth tool) was indicative of shim quality. The MGE sequence had the following parameters: TR/TE: 50.2 ms/2.82–8.77–14.72–20.67–26.62–32.57–38.52–44.47 ms (8 echoes with an echo spacing of 5.95 ms), 156 × 167 microns resolution, 1 mm axial slice, NEX = 8, acquisition time 15 min 24 s). Sequence parameter set up, acquisitions and 
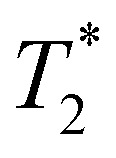
 or signal intensity analyses in regions of interest (ROIs) were performed with Image Display & Processing using Paravision (5.1 version from Bruker). For main biodistribution experiments, ROIs were placed in the liver or bone marrow in slightly *T*_2_-weighted sequences and renal pelvis in strongly *T*_2_-weighted sequences (using an external tube containing a mixture of diluted Dotarem (0.625 mM Gd in water) and D_2_O in a 1 : 3 proportion as a reference). For tumor analysis, ROIs were defined to cover the complete tumor volume visible on the selected slices.

### 2.12. *Ex vivo* immunofluorescence staining of biological tissues

Tissues were conserved in OCT (embedding matrix, Carl Roth, Karlsruhe, Germany) at −80 °C or fixed in Bouin's solution (Sigma-Aldrich, Overijse, Belgium) and embedded in paraffin (Merck Millipore, Burlington, MA, USA) for histology. The 5 μm thick slices of tumor, liver, and spleen were fixed for 10 min at room temperature with 4% PAF in PBS, then rinsed with PBS 1× and washed 3 times (10 min) with 0.3% Triton™ X-100 in PBS 1×. Next, tissues were incubated with 0.5% casein for 1 h at room temperature to block non-specific binding sites. Then, slices were incubated overnight at 4 °C with anti-PEG and anti-EGFR primary antibodies both at 1/100 dilution (see IF for cells). After 3 washes with 0.3% Triton™ X-100 in PBS 1×, tissues were incubated with the secondary antibodies, goat anti-rabbit IgG (H + L) or goat anti-mouse IgG (H + L), at 1/500 dilution for 1 h at room temperature. The slices were then rinsed with PBS 1× and incubated 15 min with Hoechst (Sigma-Aldrich, St. Louis, MI, USA), at 1 : 5000 dilution in PBS 1×. Finally, they were rinsed with PBS 1× and distilled water before being mounted with VECTASHIELD® PLUS Antifade Mounting Medium (Labconsult, Brussels, Belgium). Once dried, the slides were observed under a confocal microscope (Nikon Ti2 A1RHD25, Tokyo, Japan).

### 2.13. ICP-AES iron quantification in mouse organs

ICP-AES (Inductively Coupled Plasma-Atomic Emission Spectrometry) analysis was performed at the analytical department of the Institut Pluridisciplinaire Hubert Curien (Strasbourg, France) and under the same conditions as those in the Therapeutic Chemistry and Pharmacognosy Unit of UMONS (Mons, Belgium). For iron content, various mouse organs (liver, spleen, and tumors for mice bearing tumors) were digested in nitric acid (TraceMetal grade HNO_3_ 67–69% Fischer Chemical) at 100 °C for 2 h. Analysis of iron in the digested samples was performed by ICP-AES (Varian 720 ES). Quantification was done with a calibration curve established with standards (0, 2, 10, 50, 100, 200, and 500 μg L^−1^) prepared using a certified iron standard (1000 mg L^−1^) (CPI International).

### 2.14. Statistical analysis

Statistical analyses were performed using IBM SPSS Statistics software (version 21) (IBM, Ehningen, Germany). More than 2 independent samples were compared using an ANOVA test and a Tukey *post hoc* test. A *p*-value <0.05 was considered statistically significant (* = *p* ≤ 0.05; ** = *p* ≤ 0.01; *** = *p* ≤ 0.001). For all experiments, a minimum of 3 replicates were performed.

## 3. Results

### 3.1. Assessment of EGFR expression in a series of HNC cell lines

**Fig. 2:**
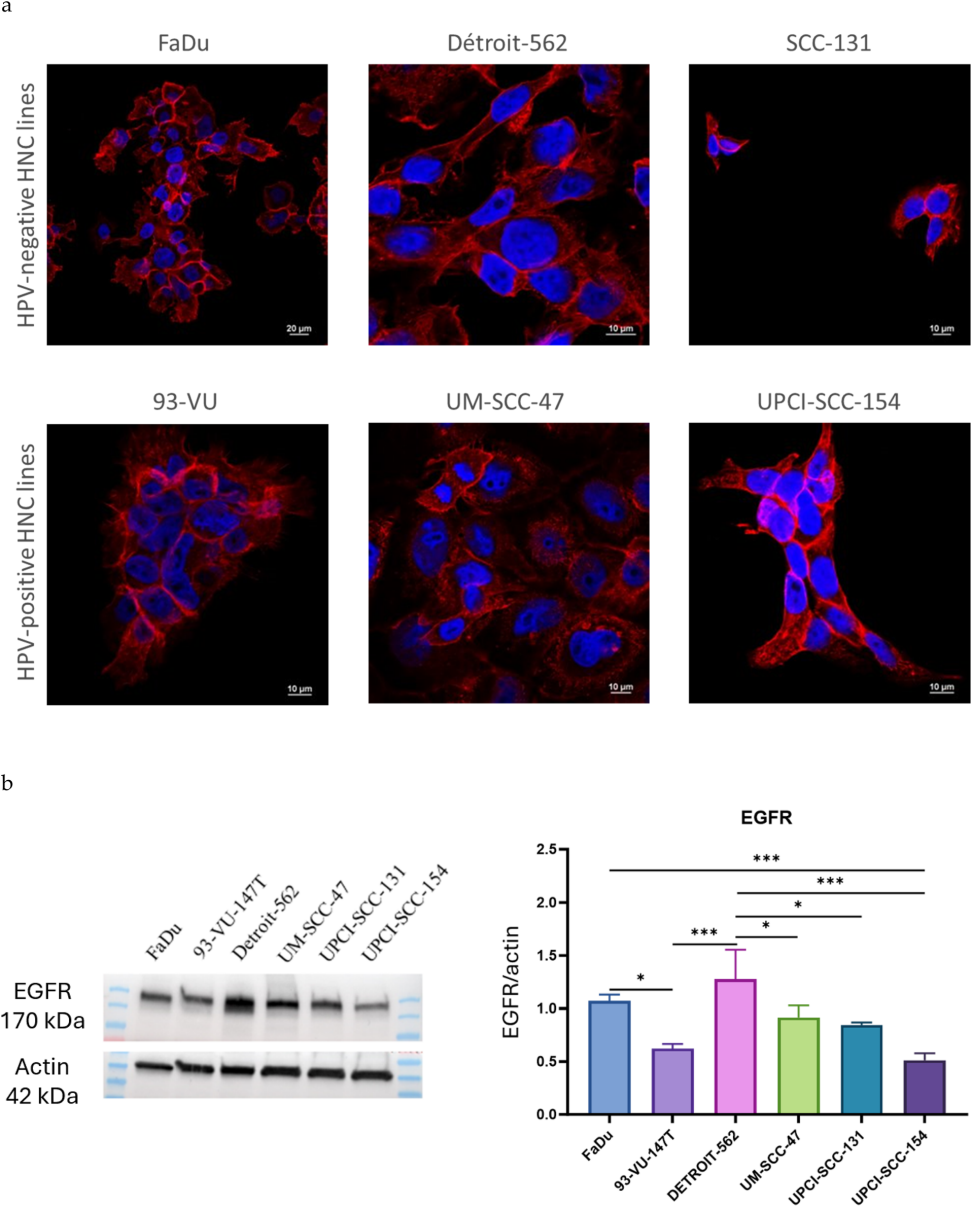
Evaluation of EGFR expression in 6 HNC cell lines. (a) Confocal microscopy images of EGFR [MA5-13269] in red, and nuclei were stained with DAPI in blue. (b) Western blot analysis (left panel) and quantification (right panel) of EGFR in the 6 lines. Molecular weight markers confirm an EGFR band at 170 kDa and actin band at 42 kDa. Specificity of the detection method was validated during the experimental phase by including a melanoma line lacking EGFR (MM074, negative control) and a breast cancer line known to overexpress EGFR (MDA-MB-231, positive control), although these data are not shown here. One-way ANOVA and a Tukey *post hoc* test were conducted; *p < 0.05; **p < 0.001; ***p < 0.001; the results present the means ± SD from three independent experiments.

Using a specific immunodetection method, we evaluated the level of EGFR expression in six HNC cell lines including three HPV negative (FaDu, Detroit-562, and SCC-131) and three HPV positive lines (93-VU, UM-SCC-47, and UPCI-SCC-154) lines. Immunofluorescence using confocal microscopy revealed significant expression of EGFR mainly in the cellular membranes in all cell lines (Fig. 2a). EGFR expression was also analyzed by western blotting and quantification revealed the highest expression of this receptor in the Detroit-562 line (Fig. 2b). As part of the experimental validation, EGFR detection was benchmarked against a melanoma line lacking EGFR expression (MM074, negative control) and a breast cancer line with high EGFR expression (MDA-MB-231, positive control), confirming the specificity of the detection method (data not shown). Based on these results, all the following *in vitro* experiments were performed using the two representative HPV− and HPV+, FaDu and 93-VU cells respectively, while the animal model was established with the FaDu line.

### 3.2. Evaluation of IONP internalization and effects on HNC cell lines

**Fig. 3:**
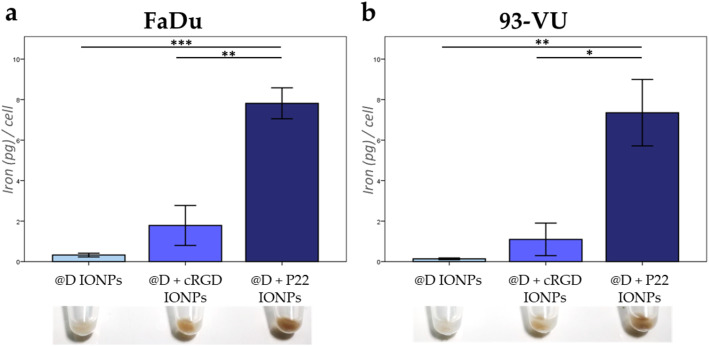
Iron content in HNC cell lines by Perls' Prussian blue colorimetric method. (a) FaDu and (b) 93-VU cells exposed for 24 h to 50 μg mL^−1^ of @D IONPs, @D + cRGD IONPs or @D + P22 IONPs. One-way ANOVA and a Tukey *post hoc* test were performed; *p < 0.05; **p < 0.001; ***p < 0.001; the results present the means ± SD from three independent experiments. Representative examples of cell pellets containing dark NPs under each experimental condition are given.

FaDu and 93-VU cell lines were exposed for 24 h to three different batches of NPs: @D IONPs, @D + cRGD IONPs and @D + P22 IONPs. For these experiments, Protocol 1 was selected as the preferred functionalization method. This approach was found to be the most effective as it allowed weak interactions between additional P22 peptides and the @D + P22 IONPs. These interactions may contribute to enhanced nanoparticle uptake. Then, NP internalization was evaluated with the Prussian blue reaction. As shown in Fig. 3, a higher amount of @D + P22 IONPs was internalized for both FaDu and 93-VU cell lines. In fact, 7.82 iron pg per cell were dosed in the FaDu line exposed to 50 μg mL^−1^ of @D + P22 IONPs compared to 0.32 iron pg per cell with @D IONPs and 1.78 iron pg per cell with @D + cRGD IONPs (*p* < 0.001 in both comparisons) (Fig. 3a). Concerning 93-VU cells, 7.35 iron pg per cell was measured in cells exposed to 50 μg mL^−1^ of @D + P22 IONPs, compared to 0.14 iron pg per cell with @D IONPs and 1.10 iron pg per cell with @D + cRGD IONPs (*p* = 0.003 and *p* = 0.012, respectively) (Fig. 3b). Each value corresponds to the mean of a minimum of three independent experiments. Of note, pellets of the cells were darker when containing higher levels of IONPs (Fig. 3a and b, bottom).

**Fig. 4:**
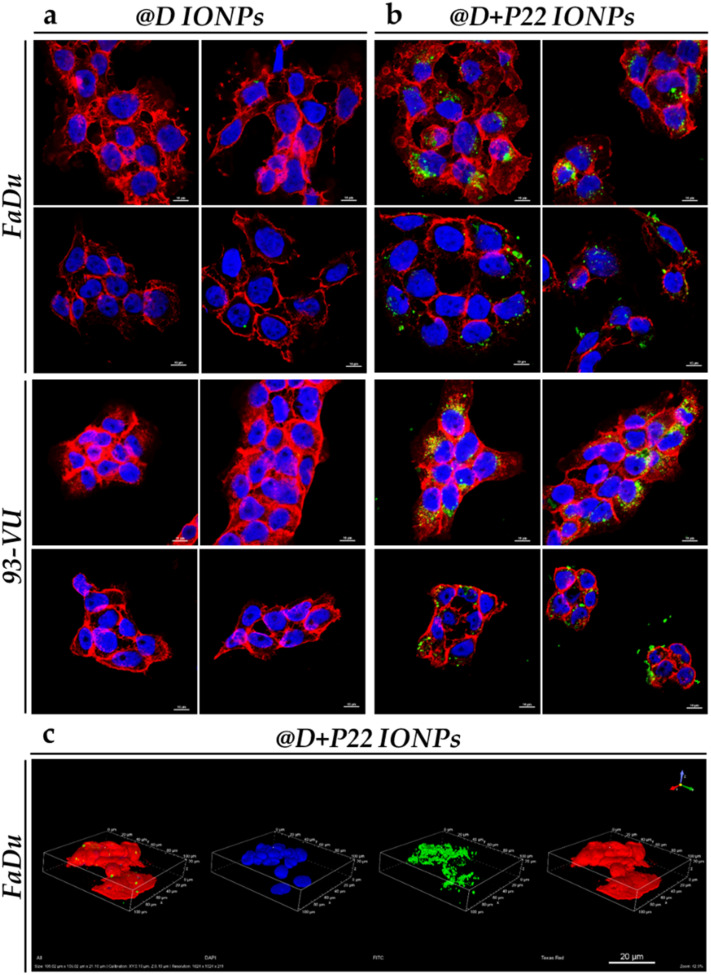
Confocal microscopy images of IONP intracellular localization in HNC cell lines. FaDu and 93-VU cells were exposed for 24 h to 50 μg mL^−1^ of (a) @D IONPs or (b) @D + P22 IONPs, scale = 10 μm. Four different pictures were presented for each condition. (c) Confocal 3-dimensional acquisition from the bottom to the top of the FaDu cell line exposed for 24 h to 50 μg mL^−1^ of @D + P22 IONPs, scale = 20 μm. Nuclei were stained with DAPI in blue, IONP clusters with anti-PEG in green and EGFR with anti-EGFR [MA5-13070] in red.

To better visualize the cellular localization of the internalized IONPs, immunofluorescence staining experiments were performed. By comparing the pictures in Fig. 4a and b, we observed intracellular IONP aggregates (in green) in FaDu and in 93-VU cell lines, only in cells exposed to @D + P22 IONPs. We again observed the homogeneous expression of EGFR (in red) in both cell lines. Moreover, the spatial distribution of the @D + P22 IONPs in FaDu cells was investigated by confocal 3-dimensional acquisition. These images showed that IONPs were not attached to the cell surface but were mainly localized in clusters in the cytoplasm and in different depths of the cells (Fig. 4c). Altogether, these data indicated that P22 vectorization improves cellular uptake of IONPs in these two HNC lines.

**Fig. 5:**
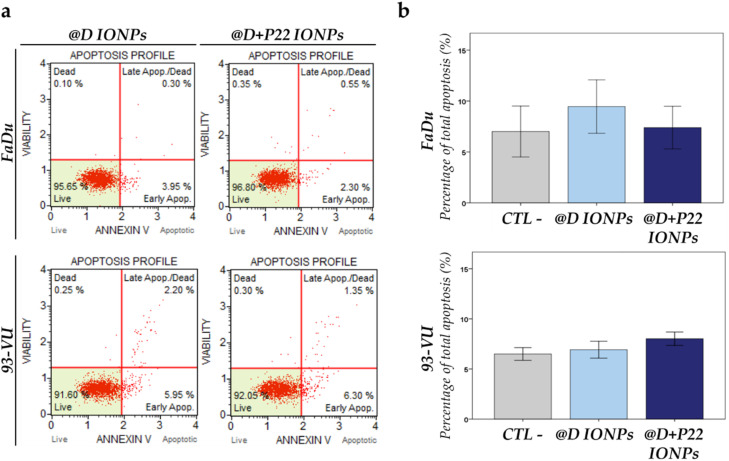
Evaluation of apoptosis in HNC cell lines induced by IONPs detected using a Muse® cell analyzer. FaDu and 93-VU cell lines were cultured for 24 h with 50 μg mL^−1^ of @D IONPs or @D + P22 IONPs. (a) Four populations of cells can be distinguished in this assay: non-apoptotic cells (live cells); early apoptotic cells; late stage apoptotic and dead cells (mostly nuclear debris). (b) Graphs represent the percentage of total apoptosis under each condition (CTL−, nontreated cells). The results were given for three independent experiments and present means ± SD. Statistical analysis did not reveal significant differences.

Next, we investigated the apoptosis level in HNC cell lines exposed for 24 h to 50 μg mL^−1^ of @D IONPs or @D + P22 IONPs. As shown in Fig. 5, apoptosis is not detected in cells when exposed to the NPs (@D IONPs or @D + P22 IONPs) (*p* > 0.05), indicating that NPs alone did not affect cancer cell survival, even if cell uptake of NPs was increased after vectorization of the nanoparticles.

**Fig. 6:**
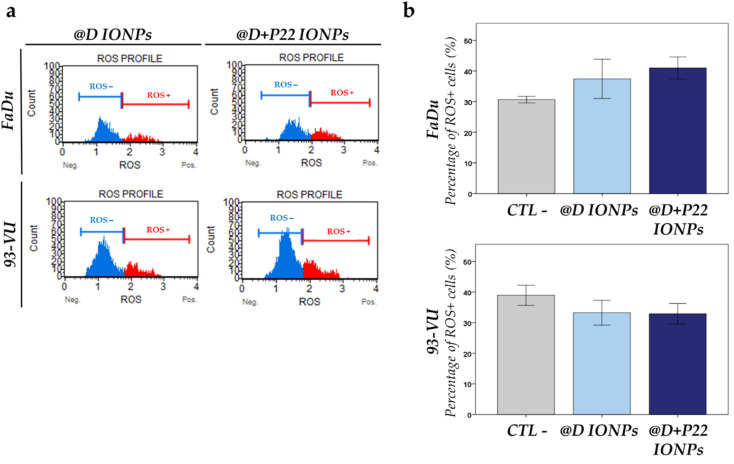
ROS evaluation after HNC cell lines were exposed to 50 μg mL^−1^ of @D IONPs or @D + P22 IONPs for 24 h. (a) Two populations can be distinguished on the graph: ROS-negative (in blue) and ROS-positive (in red) cells. (b) Graphs represent the percentage of ROS+ cells for the two cell lines when exposed to the two IONP types compared to the nontreated cells (CTL−). The results were obtained from three independent experiments and present means ± SD. Statistical analysis did not reveal significant differences.

Finally, we evaluated the oxidative stress differences and ROS levels in HNC cell lines exposed or not for 24 h to 50 μg mL^−1^ of @D IONPs or @D + P22 IONPs. In the presence of both types of NPs, no more ROS were produced compared to the control condition (cells without NPs, CTL−) (*p* > 0.05) (Fig. 6b), indicating that no oxidative stress was induced by NPs. These data were obtained using the Muse® Oxidative Stress Kit, in the form of a histogram of gated cells with the two populations (ROS− in blue and ROS+ in red) (Fig. 6a).

### 3.3. Evaluation of IONP biodistribution in animals

**Fig. 7:**
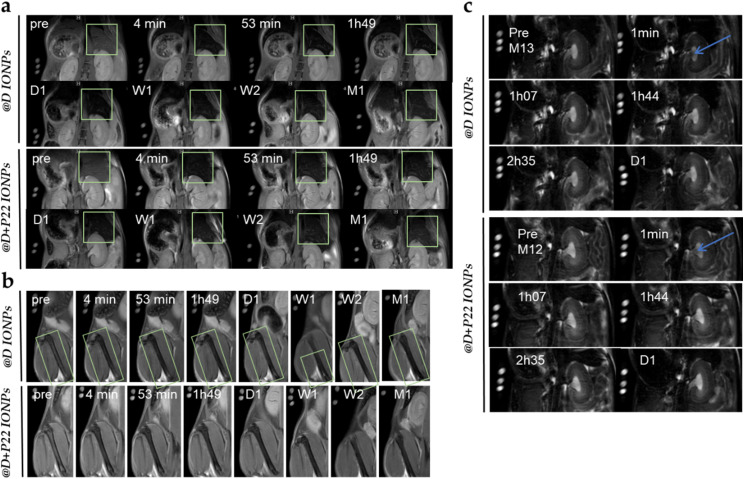
Illustration of *in vivo* MRI data in *T*_2_ contrast of mice treated with 45 μmol Fe per kg of @D IONPs or @D + P22 IONPs. Follow up of (a) liver, (b) bone marrow and (c) kidney pre-injection and at different timings as specified in the scans (D = day, W = week, and M = month). A blue arrow points at (or a green square surrounds) the area in which a change in contrast is observed.

In order to evaluate the biodistribution of our IONPs, @D and @D + P22 IONPs were intravenously injected in healthy mice at 45 μmol Fe per kg. In *T*_2_ contrast images, the liver appeared dark after 4 minutes for @D IONPs as well as for @D + P22 IONPs, reflecting IONP accumulation in organs of the mononuclear phagocytic system (MPS) (Fig. 7a). In line with the MPS uptake phenomenon, the signal of the femoral bone marrow also decreased over time (Fig. 7b). Moreover, the IONP accumulation persisted over time and was still observed 1 month post-injection, both in the liver and femur. In parallel, the signal modifications in the kidney were also investigated. The renal pelvis darkened after only 1 minute, suggesting that despite the size limitation of renal filtration, a portion of IONPs is small enough (or has an “ideal” size/charge/shape compromise) to be eliminated through the kidney at (early) times of higher circulating concentrations (Fig. 7c). In addition, daily mice follow-up did not reveal any sign of toxicity over 1 month.

**Fig. 8:**
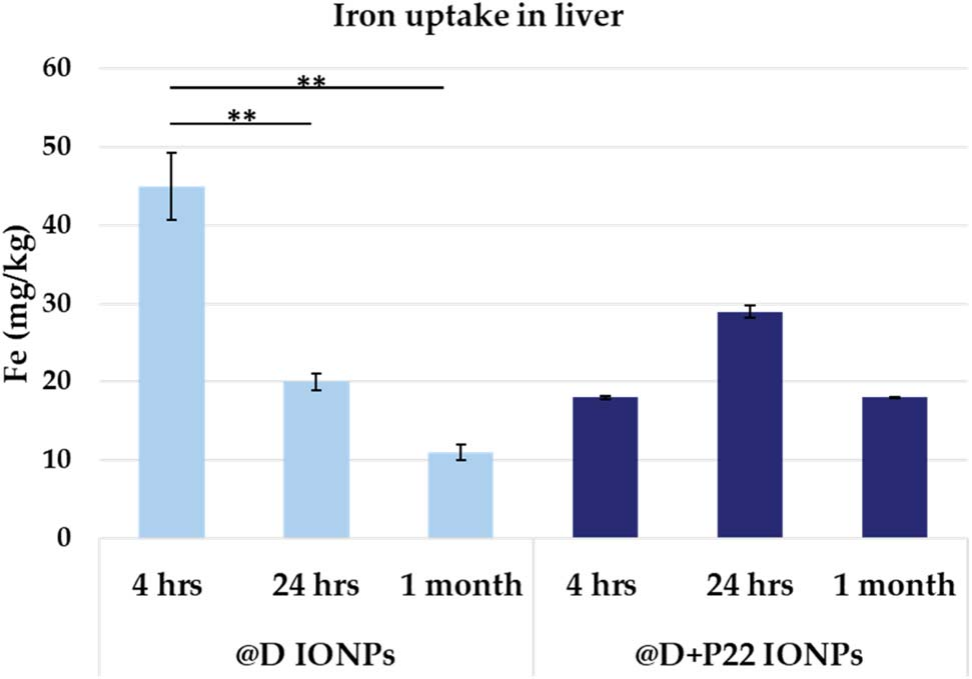
Iron uptake measured by ICP-AES analysis of livers of healthy mice (at least 3 mice per condition) 4 h, 24 h and 1 month after intravenous injection of IONPs. One-way ANOVA and the Tukey *post hoc* test were conducted; **p < 0.01; the results present the means ± SD from three independent experiments.

Moreover, to quantify the amount of IONPs accumulated in the liver of the healthy mice, ICP assessments were also performed. Four hours after IONP injection, both @D IONPs and @D + P22 IONPs accumulated in the liver. Then, for the @D IONP batch, a significant decrease was observed after 24 h and 1 month post-injection (*p* = 0.009 and *p* = 0.002, respectively). Concerning the @D + P22 IONPs, they continued to accumulate until 24 h, and then their amount tended to decrease at 1 month post-injection (data are not significant). Hence, for the two batches, after 1 month, the diminution of iron content was observed. This may be due to the clearance of NPs (also visible in MRI study), but more time should be needed to return to control values. Furthermore, a strong liver uptake is observed at 4 h post-injection for the @D + P22 IONP batch compared to the @D IONPs (Fig. 8). This might be due to its higher hydrodynamic diameter due to peptide grafting. After 1 month, there is no statistical difference between the two batches. However, the variability of the data suggests that the experiment should be repeated to have more than 3 mice per group.

**Fig. 9:**
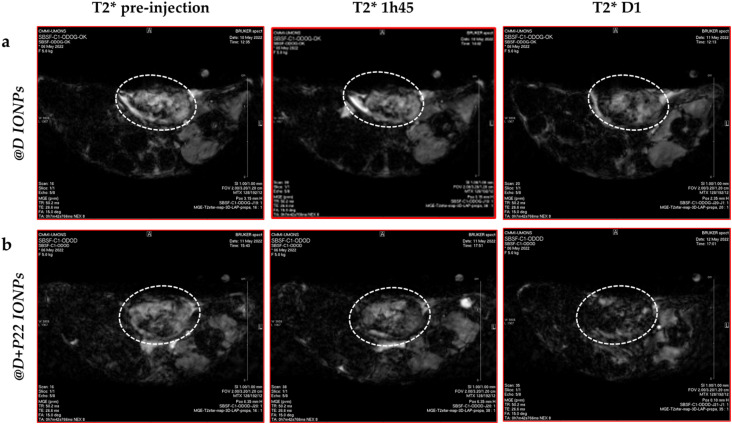
Illustration of *in vivo* MRI data of mice with tumors treated with 60 μmol Fe per kg of (a) @D IONPs (*N* = 2) or (b) @D + P22 IONPs (*N* = 4) at 
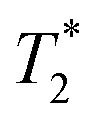
 pre-injection, 1 h 45 min and one day after injection. A dotted circle surrounds the area in which the ROI measurement was performed.

Finally, to evaluate the accumulation of our IONPs in the HNC tumors xenografted in animals, @D IONPs and @D + P22 IONPs were intravenously injected at 60 μmol Fe per kg in mice bearing a tumor from the FaDu cell line. As illustrated in Fig. 9, when comparing to the pre-injection situation on several slices, the darkening of the tumor signal was depicted by MRI at 24 h post-injection in mice injected with the @D IONPs (Fig. 9a), as well as in ones receiving the @D + P22 IONPs (Fig. 9b).

The negative contrast in tumors was assessed by 
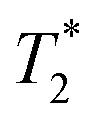
 measurements performed on the whole tumor area appearing on each slice, using the muscle surrounding the spine as a reference tissue, since it was not expected as a compartment of possible IONP accumulation. Depending on tumor size and artifacts (mainly on border (first and last) slices), 
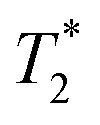
 values were measured from 9 or 10 slices and represented as a mean value before and 24 h after injection of @D IONPs or @D + P22 IONPs. 
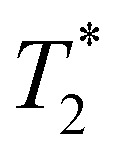
 acquisitions are highly sensitive to the presence of irregularities such as necrotic tissues and blood vessels, which have been observed on *ex vivo* tumors. These preliminary data indicated a decrease of mean 
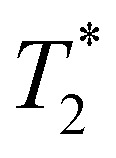
 of 10.08% in the tumor of the @D + P22 IONP injected mouse at 24 h post-injection (*N* = 4). In contrast, in the tumor of the @D IONP-injected mouse, only a slight decrease of 0.15% was observed at 24 h post-injection (*N* = 2 because of the presence of necrosis in some tumors). In the muscle, both mouse groups showed a 
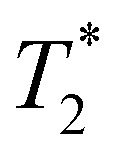
 increase of 8–9%. The reason for this reversed tendency in the muscle regarding the tumor is unknown, but this at least suggests that there was no 
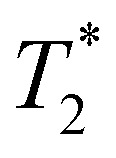
 value decrease induced by IONPs in the reference tissue. 
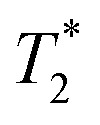
 values in the tumor were not statistically different between day 0 and day 1, but the 
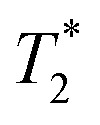
 value decrease tendency was greater with @D + P22 IONPs than with @D IONPs, offering promising perspectives for specific molecular imaging investigations in tumor models. Of note, all of these analyses were performed on the mice injected with 2.5 × 10^6^ FaDu cells. In fact, necrosis was observed in the tumor of the mice injected with 5 × 10^6^ FaDu cells, which makes this condition impossible for MRI analysis.

**Fig. 10:**
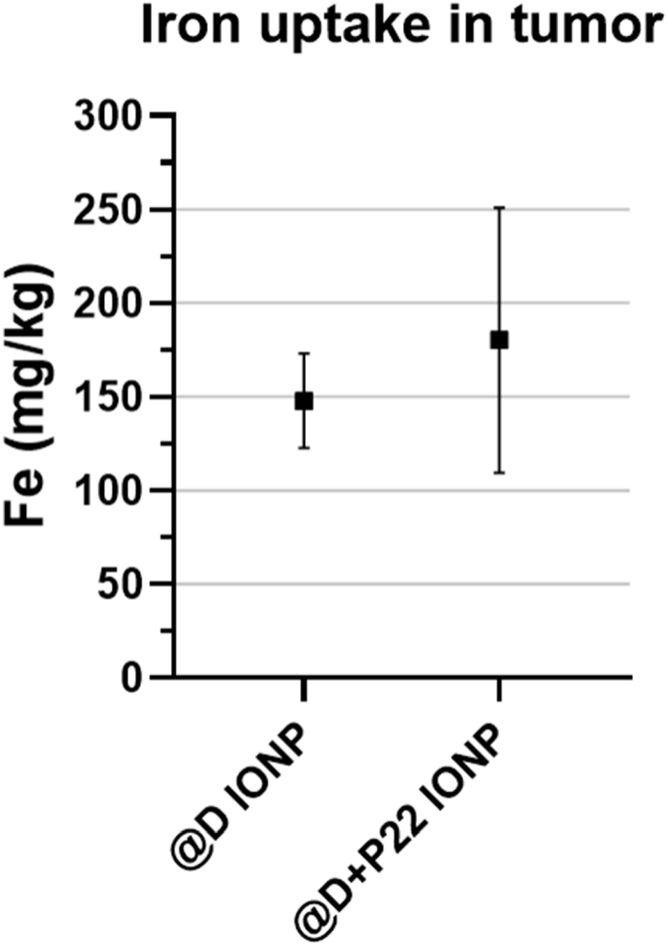
Iron uptake measured by ICP-AES analysis of tumors of mice 24 h after intravenous injection of @D IONPs (*N* = 3) or @D + P22 IONPs (*N* = 4). Statistical analysis did not reveal significant differences.

Like for the healthy mice, the amount of IONPs accumulated in the tumor of the mice, 24 h after IONP injection, was evaluated by ICP analyses. Higher iron content in tumors was highlighted, while no significant difference between IONP batches with and without P22 was detected (Fig. 10). A similar result was obtained after analysis of *ex vivo* spleen and liver iron content: no difference was shown between @D IONPs and @D + P22 IONPs.

**Fig. 11:**
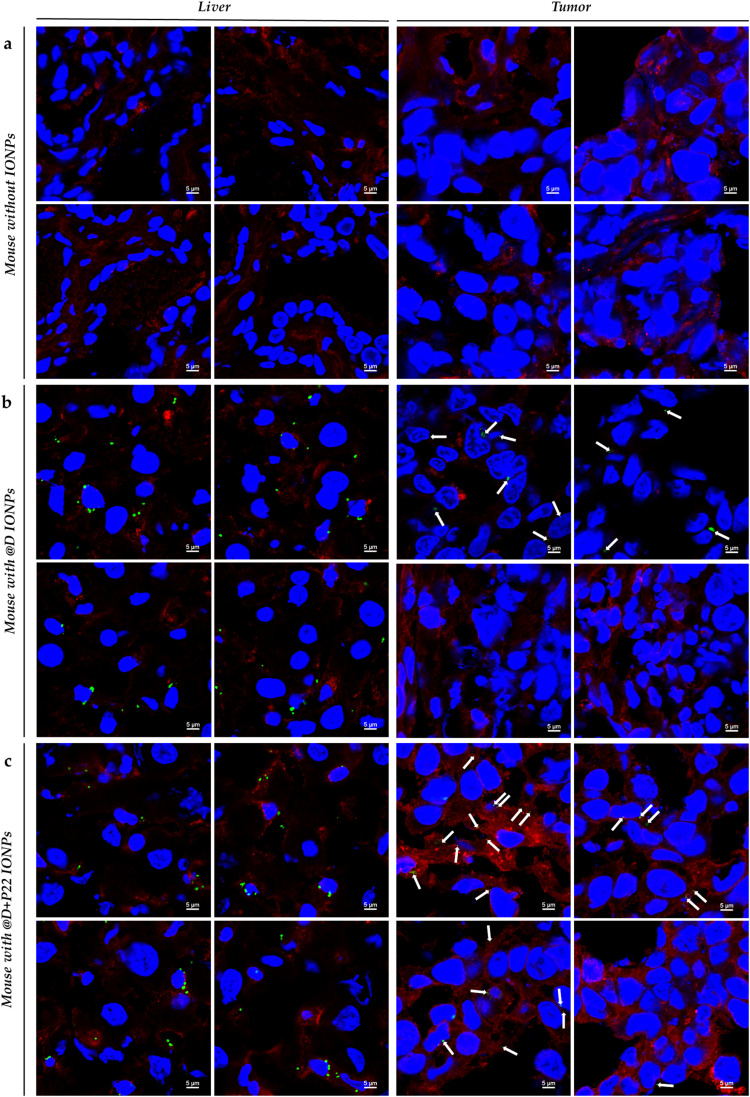
Confocal microscopy images of IONP localization in the liver and tumor of mice (a) without injection of IONPs, (b) with 60 μmol Fe per kg of @D IONPs and (c) with 60 μmol Fe per kg of @D + P22 IONPs, scale = 5 μm. Four pictures were presented for each condition. Nuclei were stained with DAPI in blue, IONP clusters with anti-PEG in green and EGFR with anti-EGFR [MA5-13070] in red.

Furthermore, in order to visualize the accumulation of IONPs, immunofluorescence staining of PEG in liver and tumor tissues was performed. By comparing the pictures in Fig. 11b and c, we observed intracellular IONP aggregates (in green) in the liver and also in the tumor of mice injected with @D IONPs and @D + P22 IONPs, compared to mice without IONPs (Fig. 11a). Undoubtedly, the accumulation of NPs in tumors was higher using @D + P22 IONPs than with @D IONPs, validating the benefit of vectorization of our NPs with P22. These images are qualitative, intended to illustrate tissue localization of IONPs, and were not processed with standardized exposure or quantitative ROI-based analysis. Accordingly, they should not be interpreted as quantitative comparisons of uptake between the tumor and liver. Quantitative assessments of biodistribution are provided by MRI and ICP-AES.

## 4. Discussion

Our results clearly demonstrate that it is essential to vectorize IONPs to improve their internalization in HNC cell lines. In fact, @D IONPs were less internalized by FaDu and 93-VU cell lines than @D + P22 IONPs, as shown by Perls' Prussian blue colorimetric dosage and immunofluorescence. Non-vectorized IONPs are coated with dendron molecules which are composed of a biphosphate group, allowing anchoring the iron and three polyethylene glycol (PEG) chains with the middle one, which is longer and bears a functional end group allowing it to link molecules. Also, the chains of PEG prevent NPs from aggregating.^25,26^ In order to increase the IONP internalization rate, we grafted cRGD motifs on these NPs like in previous study. Indeed, this model is very well-documented in the literature and proven to promote NP capture by cancer cells.^14^ Despite the fact that HNC cell lines are reported to express high levels of αvβ3 and αvβ5 integrins, known to allow binding to the RGD motif, a low amount of @D + cRGD IONPs was internalized by our FaDu and 93-VU cell lines.^15^ For the FaDu cell line, this may be explained by the fact that these cells would not express αvβ3 integrins.^27^ Consequently, another IONP model was synthesized using P22 for @D IONP vectorization to the EGFR. Indeed, this peptide has a high affinity for the EGFR, as reported by Hossein-Nejad-Ariani *et al.*^18^ First, we demonstrated that HNC cell lines expressed high levels of EGFR. Then, in our model using FaDu and 93-VU lines, a significantly high quantity of @D + P22 IONPs was internalized by the cells. However, our internalization experiments did not include an EGFR-low or EGFR-negative control line, nor did we perform competition assays with free EGF, which would be required to firmly establish receptor-mediated uptake. Our assessment of specificity was instead based on confirming EGFR expression by western blot and quantifying nanoparticle uptake in two EGFR-positive lines. Although 93-VU cells showed lower total EGFR levels than FaDu cells, uptake was comparable in both lines, which may reflect differences between total and membrane-localized receptor pools or the involvement of alternative endocytic pathways. These limitations are acknowledged as important directions for future studies, where additional negative control models (EGFR silencing and additional lines) and competition experiments will be necessary to validate EGFR-specificity. This suggests that vectorized NPs are a good candidate for applications requiring accumulation of nanomaterials in HNC tumors. Of note, cell apoptosis and ROS levels were evaluated when HNC cells were exposed to these IONPs, and as expected, no more apoptosis or ROS production was detected in the presence of NPs. These results suggest that these NPs alone are not able to affect cancer cells and probably not normal cells, avoiding side effects. This point is very beneficial for cancer treatment as it is possible to combine different treatments such as local radiation therapy with tumor targeted NPs used as radiosensitizers.^28^ Indeed, the efficacy of IONPs as radiosensitizers is well-demonstrated in the literature, reporting that the most important challenge is the specific internalization of NPs in cancer cells.

Then, our IONP model was evaluated using an *in vivo* model to assess the biodistribution in healthy tissues. Once in circulation, NPs are very rapidly eliminated by phagocytic monocytes and by the reticuloendothelial system (RES) and accumulate mainly in the liver and in the spleen. This was investigated by MRI because IONPs represent an excellent 
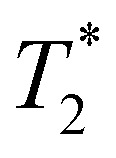
 contrast agent.^29^ In light of these results, it should be important to further examine whether morphological changes or cytotoxic effects are detected in the liver and/or spleen after IONP accumulation for a long term. Interestingly, our results highlight that our NPs do not induce toxicity until 1 month post-injection. However, it would be interesting to investigate the possible histological changes by hematoxylin and eosin staining in future studies.

Next, our work focused on the demonstration of the intratumoral accumulation of the vectorized IONPs. Indeed, in the case of tumor targeting, it is also important to consider the tumor microenvironment because vascularization within a tumor is very heterogeneous and abnormal (neo-angiogenesis). Besides, the pressure of the interstitial fluid is increased, due to the obstruction by the tumor, which also impacts the accumulation of NPs. Moreover, the distribution and the passive accumulation of NPs within tumors are accentuated by the “enhanced permeability and retention” (EPR) effect, in which the increase in vascular permeability to particles may be due to large fenestrations between endothelial cells, and the intra-tumor accumulation of particles may arise from defective lymphatic drainage.^30^ However, this EPR effect is highly variable between patients and depends in particular on the tumor stage. In fact, the EPR effect is greater in early stage tumors compared to advanced stage tumors where blood vessels are occluded or embolized.^31^ In order to compensate for this interindividual tumor heterogeneity and to favor active targeting, the P22 ligand may help increase the intra-tumoral accumulation of IONPs. Indeed, concerning our *in vivo*–*ex vivo* studies on the tumor model, we have collected results of MRI, ICP-AES and immunofluorescence that suggest IONP accumulation in the tumor. However, the confocal images (Fig. 11) should be interpreted only as qualitative. They were acquired to visualize the localization of PEG-labeled IONP clusters in liver and tumor tissue and to provide descriptive support to the MRI/ICP-AES data. Any difference suggested by these images should therefore be considered descriptive only, while quantitative conclusions are based on MRI and ICP-AES. Our preliminary data indicate that @D + P22 IONPs tend to be more accumulated in mouse tumors than @D IONPs, even if significance was not reached in some experiments. However, these results are based on a very small number of mice, and they should be further validated. In fact, some aberrant results were obtained with mice injected with 5 × 10^6^ FaDu cells, which were excluded due to necrosis within these large tumors. In the literature, it is reported that when an FaDu subcutaneous tumor reaches 100 mm^3^ in size, the tumor is already hypoxic. This was highlighted by Chen *et al.* who investigated the effect of the oxygen microenvironment on IONP uptake in HNC cell lines. Indeed, they showed that IONPs are more taken up by cells under hypoxic conditions, in an *in vitro* as well as in an *in vivo* model. This may be explained by the morphological change of the cells under hypoxia.^32^ However, in this study, IONPs were injected directly into the tumor, and therefore it is not completely comparable to our *in vivo* model, in which IONPs were intravenously injected. In contrast, it is also documented that IONPs had poor uptake in cells which were exposed to hypoxia. Indeed, the NPs alone are not able to diffuse into the hypoxic and necrotic areas of the tumor because there is no/low vascularization. Yang and his colleagues showed that the use of macrophages as a transporter allows for better diffusion of NPs within hypoxic tumor tissue.^33^ In this context, it should be important to investigate the capacity of macrophages to internalize and transport IONPs, based on our previous work.^34^ To conclude, our IONP model vectorized with P22 seems to be appropriate to achieve internalization in HNC cell lines overexpressing EGFR. More analyses would be performed to study the interest of the combination with other treatments, such as radiotherapy, since IONPs can act as radiosensitizers. Altogether, these preliminary results are encouraging and open some relevant perspectives in the field of target nanomedicine.

## Author contributions

The manuscript was written through contributions of all authors. The experiments were mainly designed by S. S., S. B.-C., F. J., and S. L. and performed by S. F., T. G., B. F., M. L. A. R., G. D., D. S., S. B., L. L. and A. T. The manuscript was mainly written by S. F., T. G. and S. L. as main contributors to the texts. S. F., T. G., B. F., M. L. A. R., A. T. and G. D. designed and performed cell culture experiments and related analyses. S. B. and L. L. performed the imaging studies. All authors have given approval to the final version of the manuscript.

## Conflicts of interest

There are no conflicts to declare.

## Supplementary Material

NA-007-D5NA00361J-s001

## Data Availability

All relevant data supporting the findings of this study are presented in the article. The raw datasets were generated independently by multiple laboratories and are not publicly available in a centralized repository. However, they remain accessible within the respective research institutions and can be provided upon reasonable request, subject to institutional policies and agreements. Datasets from *in vitro* experiments can be found at the Department of Human Anatomy and Experimental Oncology, Faculty of Medicine, Research Institute for Health Sciences and Technology, University of Mons. Datasets from *in vivo* experiments can be found at the Center for Microscopy and Molecular Imaging, B-6041 Gosselies, Belgium, and General, Organic and Biomedical Chemistry Unit, NMR and Molecular Imaging Laboratory, University of Mons, B-7000 Mons, Belgium. Supplementary information is available: Full uncropped and unprocessed western blot images corresponding to Fig. 2 (anti-EGFR antibody, and anti-actin antibody). See DOI: https://doi.org/10.1039/d5na00361j.
