# Supplementary material for: In vitro and in vivo validation studies of optimized iron oxide nanoparticles carrying targeting ligands for a new therapeutic strategy in head and neck cancers
Source: Nanoscale Adv. 2025 Sep 3;7(21):6987–7002. doi: 10.1039/d5na00361j (PMC12455219; doi:10.1039/d5na00361j)
Supplement: NA-007-D5NA00361J-s001 [file NA-007-D5NA00361J-s001.pdf]

Supplementary Information (SI) for Nanoscale Advances.
This journal is © The Royal Society of Chemistry 2025

Supplementary data

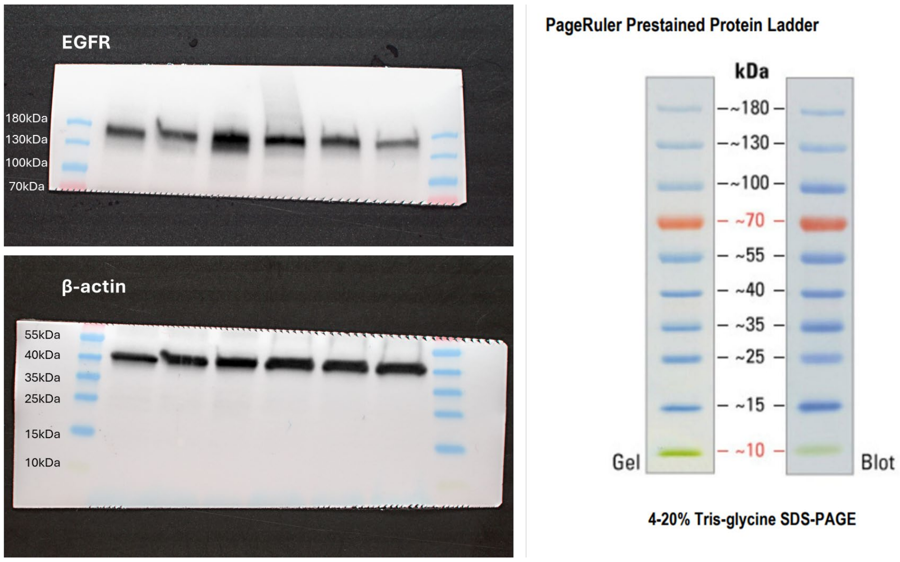

Supplementary Figure 1:

Full, uncropped and unprocessed western blot images corresponding to figure 2. After electrophoresis and transfer onto the membrane, the membrane was cut at the 70 kDa marker. The upper part was used with the anti-EGFR antibody, and the lower part with the anti-actin antibody (43 kDa) for loading control check. The membrane was cut to allow incubation of the two antibodies in parallel and simultaneously, each on their respective part of the membrane.
